# Prevalence of prehypertension and hypertension among the adults in South Asia: A multinomial logit model

**DOI:** 10.3389/fpubh.2022.1006457

**Published:** 2023-01-27

**Authors:** Dil Bahadur Rahut, Raman Mishra, Tetsushi Sonobe, Raja Rajendra Timilsina

**Affiliations:** ^1^Asian Development Bank Institute (ADBI), Tokyo, Japan; ^2^Population Council Consulting, New Delhi, India

**Keywords:** prehypertension, hypertension, multinomial logit model, South Asia, cardiovascular risk

## Abstract

Hypertension has been the most common non-communicable disease in low and middle-income countries for the past two decades, increasing cardiovascular and renal disease risk. Urbanization, aging, dietary and lifestyle changes, high illiteracy rates, poor access to health facilities, poverty, high costs of drugs, and social stress have contributed to an increase in the prevalence of hypertension in developing countries. Nonetheless, little is known about the comprehensive risk factors associated with prehypertension and hypertension among economically active adult populations of South Asia, such as India, Nepal, and Bangladesh. This paper uses the Demographic and Health Survey data of 637,396 individuals from India (2019–21), 8,924 from Nepal (2016), and 8,613 from Bangladesh (2017–18) to examine the prevalence and driver of prehypertension and hypertension. We analyze the prevalence of prehypertension because it leads to hypertension and is directly related to cardiovascular disease, and many people live with it for prolonged periods without realizing it. The paper finds, among other things, that the prevalence of prehypertension and hypertension among adults (18–49 years) is 43.2 and 14.9% in India, 35.1% and 19.8% in Bangladesh, and 25.2% and 13.8% in Nepal, respectively. Better educated, wealthy individuals living in urban areas of developing economies in the South Asian region are more likely to have prehypertension and hypertension. The paper suggests the urgent need to launch preventive programs to reduce prehypertension before it develops to be hypertension as a precautionary measure. Thus, such measures shall help to prevent hypertension, thereby improving the overall wellbeing of individuals and families.

## 1. Introduction

Hypertension, also known as cardiovascular disease, accounts for most non-communicable disease deaths globally (77%) (1). Hypertension has become a significant public health challenge and is one of the leading risk factors for death in low- and middle-income countries. Hypertension is prevalent among older adults and ubiquitous among younger ages too. The Seventh Joint National Committee on Prevention, Detection, Evaluation, and Treatment of High Blood Pressure first coined the word “prehypertension” as one of the categories for blood pressure in 2003 (2). Prehypertension is a precursor of hypertension; independently, it has a strong positive linear relationship with “cardiovascular disease (CVD)” morbidity and mortality. Several studies have also claim an association between prehypertension and chronic kidney diseases (3, 4). Prehypertension leads to hypertension eventually with an increase in age, and the share of the young population with prehypertension is growing in low-and middle-income countries (5).

Globally, hypertension affects approximately one in four adults, and the number of adults with hypertension in 2025 is predicted to increase by about 60% to a total of 1.56 billion population (6). Cardiovascular risk factors start early, track through a young age, and manifest in middle age in most societies (7). However, the comprehensive study of risk factors associated with prehypertension and hypertension in an economically active or industrial population of South Asian countries, such as India, Nepal, and Bangladesh, is rare. Thus, this study uses the recent Demographic Health Survey (DHS) data from India (2019–2021), Nepal (2016), and Bangladesh (2017–18) to deepen the understanding of the prevalence of prehypertension and hypertension and the factor associated with its prevalence. More specifically, data on socioeconomic, geographical, and risk factors associated with prehypertension and hypertension among adults (18–49 years old) are examined by employing multinomial logit models across the three countries. The contribution of this paper is that it explores both prehypertension and hypertension situations in South Asia, which has not been as systematically and rigorously explored in the previous literature. Further, it uses the most recent 2019–21 DHS data for India.

## 2. Literature review

Several low-and middle-income countries are undergoing an epidemiological transition and the rising burden of non-communicable diseases (NCDs)^1^. For instance, Naghavi et al. (9) estimate the cause-specific deaths and years of life lost by age, sex, geography, and year and report that NCDs represent 72.3% of total deaths. Among the NCDs, hypertension is one of them, and past studies find several factors explaining the prevalence of hypertension in Asian and South American countries [e.g., (10)]. A study in Brazil using data from six Brazilian state capitals finds that males are more hypertensive than females, and a lower percentage of hypertension was recorded among the control participants with undergraduate compared with postgraduate university education level (11). Using a cross-sectional population-based national survey data from Indonesia, Peltzer and Pengpid (12) find a high prevalence of hypertension among the general adult population and that while more than half of hypertensive patients are aware of hypertension, only a small minority are aware of the risk factors such as obesity, physical inactivity, and stress. Based on the *Demographic and Household Survey Data 2016*, Hasan et al. (13) find that hypertension is higher among wealthy, overweight/obese, older, and educated individuals living in urban areas in Nepal. Consumption of unhealthy products, such as alcohol, sugar and sugar-sweetened beverages (SSBs), tobacco, salt, and junk foods, are significant determinants of NCDs such as heart disease and stroke due to hypertension (14–17). Using Ministry of Health and Population (MoHP) (18), Agho et al. (19) find that prehypertension and hypertension are higher in males than females. Overall, there are several significant modifiable determinants of prehypertension and hypertension, such as unhealthy habits, obesity, tobacco smoking, and alcohol, and some of these are gender specific.

Basu et al. (20) perform a mean prevalence simulation study on SSBs-related fiscal policies in India with the general population to find that a 20% increase in SSBs excise tax could reduce overweight and obesity prevalence by 3% between 2014 and 2023. Sanchez-Romero et al. (21) conduct a study in Mexico with adults aged 35–94 years and suggests that an excise tax increase by 10% would result in about 189,300 fewer incidents of type 2 diabetes cases and 20,400 fewer incidents of fatal strokes between 2013 and 2022. Barrientos-Gutierrez et al. (22) simulate the SSBs tax policy in Mexico for people aged above 20 years and find that it could prevent 86,000–134,000 cases of diabetes by 2030. Overall, SSB taxation at a high rate is a viable solution to mitigate rising obesity, type 2 diabetes, and CVD among developing economies.

Sharma (23) argue in his review article that South Asian countries (India, Pakistan, Bangladesh, Sri Lanka, and Nepal) are emerging as the epicenter of the epidemic of hypertension, and this large subcontinent within Asia shares geographic boundaries and economic and cultural similarities. Gupta et al. (7) conduct population-based epidemiological studies to identify cardiovascular risk factors in North India from 1999 to 2002. Authors claim that hypertension is prevalent in adolescents, and rapid escalation of these risk factors by the age of 30–39 years is noted among urban Indians. Jeemon et al. (24) analyze data from a nationwide surveillance program in India and conclude that the Indian industrial population shows that cardiovascular risk factors clustered with elevated blood pressure and hypertension and are at significantly,” increased heart disease risk. Overall, the socioeconomic gradient in cardiovascular diseases and related risk factor distributions among population groups are also changing, such that hypertension is positively associated with higher socioeconomic status in both Indian urban and rural areas (25).

Studies using Bangladesh Demographic and Health Survey (BDHS) find that a large proportion of adults aged 18–34 are hypertensive in Bangladesh (5, 26). Using data from the BDHS 2011, (27, 28), confirm that the risk of hypertension is significantly associated with age, gender, education, place of residence, wealth index, BMI, and diabetes. Chowdhury et al. (29) conduct a systematic review and meta-analysis and report that hypertension is rising in Bangladesh and is generally asymptomatic, and people are unaware that they have it.

Therefore, it is vital to look at prehypertension and hypertension together in South Asia among economically active population groups as the prevalence is high, remains unknown, and affects the most productive age group. The past literature has explore the prevalence of hypertension various and the factors associated with hypertension (11–13). Studies have also stress the urgency to bring policy programs, such as awareness programs, and interventions, for controlling food habits, tobacco and alcohol use, and taxes to effectively reduce the burden of NCD in middle and low-income countries (1). High illiteracy rates, poor access to health facilities, bad dietary habits, poverty, and high costs of drugs are found to be accompanied by low funds, poor infrastructure, and inexperience, contributing to the prevalence of hypertension [e.g., (30)]. Understanding prehypertension and hypertension in South Asian countries with economically active populations is vital as that will directly affect the country's socioeconomic development. Thus, all these results of the existing studies mentioned above are informative and provide a guide in designing our research that explores socio-demographic and other characteristics associated with prehypertension and hypertension in a unified framework to develop preventive programs to stop prehypertension from becoming hypertension.

## 3. Methodology

### 3.1. Data source

This study uses the nationally representative data collected through a recent round of DHS in India (2019–2021), Nepal (2016), and Bangladesh (2017–2018). The data collection applied a multi-stage sampling process. The enumeration areas and the primary sampling units were selected in the first step. In the second stage, sample households were selected from the primary sampling units. As a result, 637,396 respondents in India, 8,924 in Nepal, and 8,613 in Bangladesh, aged 18–49 years, were selected for blood pressure measurement. Individuals on medication for hypertension were excluded from the analysis, and other individuals with the missing outcome and dependent variables were also excluded from the study. Details about the data collection process are available on the DHS website. The ethical review of the data collection was done by the DHS and the government of the country involved.

### 3.2. Data measurement

The DHS collects three systolic and diastolic blood pressure readings from all eligible respondents aged 18–49 years. The second and third readings of each type of blood pressure are averaged out to represent the respondent's systolic or diastolic blood pressure. The dependent or response variable in our empirical model has three categories: “*normal*,” “*prehypertensive*,” and “*hypertensive*.” Respondents with systolic and diastolic being 140 mm Hg over and 90 mm Hg over, respectively, are considered hypertensive. Those respondents with systolic between 130 and 140 or with diastolic between 80 and 90 are classified as prehypertensive. Explanatory variables were selected based on the literature review and the data availability across the three countries. The explanatory variables used in the models include age, sex, place of residence, education, income level, wealth status, marital status, and body mass index (BMI) (see Table 1 for the explanation of the variable used in the study).

**Table 1:** Explanation of variable used in the study.

**Outcome**	**Definition**
Hypertension	Respondents having systolic and diastolic being 140 mm Hg over and 90 mm Hg over, respectively, are hypertensive.
Prehypertension	Those respondents with systolic between 130 and 140 or with diastolic between 80 and 90 are classified as prehypertensive.
Normal	Those respondents having systolic below 130 and diastolic below 80 are considered in normal blood pressure
**Explanatory**	
**Age (years)**	
18–30 ^(a)^	Age of the respondent 18 yrs and above and 30 yrs. and below
31–39	Age of the respondent 31 yrs and above and 39 yrs. and below
40–49	Age of the respondent 40 yrs and above and 39 yrs. and below
**Sex**	
Male^a^	If the respondent is male
Female	If the respondent is female
**Place of residence**	
Urban^a^	If the respondent is residing in an urban location
Rural	If the respondent is residing in a rural location
**Educational level**	
Illiterate^a^	If the respondent is illiterate
Primary	If the respondent has completed the primary level of education
Secondary	If the respondent has completed a secondary level of education
Higher	If the respondent has completed a higher level of education
**Wealth index**	
Poor^a^	Poor was computed by combining Poor and poorer
Middle	Middle was kept as middle
Rich	Rich was computed by combining Rich and Richer
**Marital status**	
Never married^a^	If the respondent has never married
Currently married	If the respondent has currently married
Formerly/ever married	If the respondent has formally married
**Body mass index**	
Normal^a^	If BMI is 18.5 to < 24.9, it falls within the Normal weight range.
Overweight	If BMI is 25.0 to < 30, it falls within the overweight range.
Obese	If BMI is 30.0 or higher, it falls within the obesity range.

a Used as a base category in the regression model.

## 4. Empirical model

Descriptive and econometric analyses are done separately for each country, and accordingly, tables reporting descriptive statistics and regression coefficient estimates are provided independently for India, Nepal, and Bangladesh. Since the dependent variable is three discrete and mutually exclusive, multinominal logistic regression is used to understand the factors associated with prehypertension and hypertension among adults aged 18–49 years.

We estimate a set of coefficients, β^(1)^, β^(2)^, and β^(3)^ corresponding to each outcome in the multinomial logit regression model,


$$\begin{array}{l} P_{1}= Pr\left(y=1\right)=\;\frac{e^{X\beta^{\left(1\right)}}}{e^{\beta^{\left(1\right)}}+e^{\beta^{\left(2\right)}}+e^{\beta^{\left(3\right)}}}\\ P_{2}= Pr\left(y=2\right)=\;\frac{e^{X\beta^{\left(2\right)}}}{e^{\beta^{\left(1\right)}}+e^{\beta^{\left(2\right)}}+e^{\beta^{\left(3\right)}}}\\ P_{3}= Pr\left(y=3\right)=\;\frac{e^{X\beta^{\left(3\right)}}}{e^{\beta^{\left(1\right)}}+e^{\beta^{\left(2\right)}}+e^{\beta^{\left(3\right)}}} \end{array}$$

The model, however, is unidentified in the sense that there is more than one solution to β^(1)^, β^(2)^, and β^(3)^ that leads to the same probabilities for *y* = 1, *y* = 2 and *y* = 3


$$\begin{array}{l} P_{1}+\;P_{2}+\;P_{3}=1 \end{array}$$

Where,

*P*_1_ = the estimated probability of normal blood pressure.

*P*_2_ = the estimated probability of prehypertension.

*P*_3_ = the estimated probability of hypertension.

The relative probability of *y* = 2 to the base outcome *i*.


$$\begin{array}{l} \frac{P_{2}}{P_{1}}=\frac{\mathrm{Pr}\;\left(y=2\right)}{\mathrm{Pr}\left(y=1\right)}=e^{{X\beta }^{2}} \end{array}$$

Thus, the exponentiated value of the coefficient is the relative-risk ratio for a one-unit change in the corresponding variable.

## 5. Empirical results

### 5.1. Descriptive statistics

**Figure 1:**
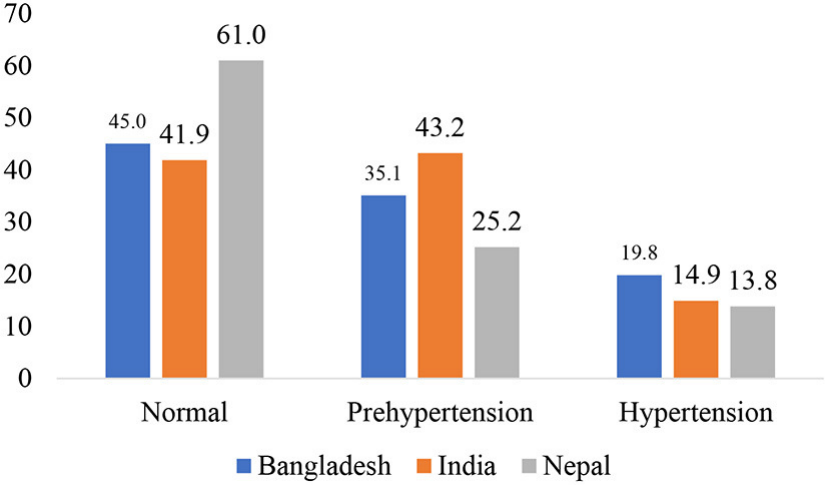
Prevalence of prehypertension and hypertension among adults (18–49 years) in South Asia.

Figure 1 shows the prevalence of prehypertension and hypertension among adults (18–49 years) in South Asia. In Bangladesh, 45% of adults has normal blood pressure and 35.1% are prehypertensive, and 19.8% are hypertensive. In the case of India, 41.9% had normal blood pressure and 43.2% are prehypertensive, and 14.9% are hypertensive. In Nepal, 61.0% has normal blood pressure, 25.2% are prehypertensive, and 13.8 are hypertensive.

Table 2 shows the descriptive statistics by country and blood pressure status (normal, prehypertensive, and hypertensive), which is used as the dependent variable in the empirical model. The percentage of households with normal blood pressure for the age group 18–30 years, 31–39 years, and 40–49 years are 53.2, 36.4, and 26.4% in India, 73.1, 53.6, and 42.9% in Nepal and 54.5, 38.5, and 32.1% in Bangladesh. It is evident from Table 1 that in India, Nepal, and Bangladesh, closer to 50% of the economically active young population have normal blood pressure. It is also clear that normal blood pressure percentage declines with the increase in the age group. All three countries' cohorts of 18–30 years have a smaller percentage of hypertension compared with other groups, and hypertension percentage increases in the cohort of the age group of 31–39 years and 40–49 years. Interestingly, the prehypertension percentage among 18–30 very young adults in India is 39%, 20% in Nepal, and 34 % in Bangladesh. India has the highest percentage of prehypertension in all three age cohorts: 18–30, 31–39, and 40–49.

**Table 2:** Prevalence of prehypertension and hypertension by background characteristics in South Asia.

**Characteristics**	**Bangladesh (*****N =*** **8,613)**			**India (*****N =*** **637,396)**			**Nepal (*****N =*** **8,924)**		
	**Normal**	**Pre-hypertensive**	**Hypertensive**	**Normal**	**Pre-hypertensive**	**Hypertensive**	**Normal**	**Pre-hypertensive**	**Hypertensive**
**Age (years)**									
18–30	54.5	34.8	10.7	53.2	39.2	7.6	73.1	20.6	6.3
31–39	38.5	36.7	24.8	36.4	47.1	16.5	53.6	29.6	16.8
40–49	32.1	34.0	33.9	26.4	46.9	26.7	42.9	30.2	26.9
**Sex**									
Male	40.9	41.0	18.0	31.0	51.3	17.7	50.7	30.8	18.4
Female	47.7	31.3	21.0	50.7	36.7	12.6	67.5	21.6	10.9
**Place of residence**									
Urban	43.2	35.3	21.5	39.8	44.0	16.3	60.4	24.9	14.6
Rural	45.8	35.1	19.2	42.9	42.9	14.2	61.9	25.6	12.4
**Educational level**									
Illiterate	39.7	36.7	23.6	39.0	43.7	17.3	60.7	26.4	12.9
Primary	46.0	34.2	19.8	38.7	44.3	17.0	59.0	25.1	15.8
Secondary	46.6	34.8	18.6	42.7	42.8	14.5	60.8	24.9	14.4
Higher	45.1	36.0	18.9	44.5	43.1	12.3	63.6	24.3	12.0
**Wealth index**									
Poor	48.8	34.8	16.4	44.6	42.4	13.1	59.8	27.4	12.8
Middle	43.7	37.0	19.4	42.2	42.8	15.0	63.6	24.5	11.9
Rich	42.2	34.5	23.2	39.3	44.2	16.5	60.7	23.8	15.5
**Marital status**									
never married	50.6	38.4	11.0	50.4	41.5	8.1	72.1	22.5	5.3
currently married	44.2	34.8	21.0	39.5	43.8	16.6	59.0	25.6	15.4
formerly/ever married	43.2	30.9	25.9	36.6	41.7	21.7	52.5	30.8	16.6
**Body mass index**									
Normal	50.7	34.9	14.4	52.5	37.3	10.2	66.2	23.7	10.2
Overweight	26.0	37.7	36.3	35.0	43.9	21.1	41.8	32.8	25.5
Obese	18.3	41.5	40.2	28.0	42.4	29.6	31.7	31.5	36.9
**Total**	45.0	35.1	19.8	41.9	43.2	14.9	61.0	25.2	13.8

Source: authors' estimation from DHS data.

Prehypertension is higher among males than their female counterparts: 51.3 vs. 36.7% in India, 30.8 vs. 21.6% in Nepal, and 41.0 vs. 31.3% in Bangladesh. Except for Bangladesh (18.0 vs. 21.0%), a similar trend for hypertension among males and females is found in India (17.7 vs. 12.6%) and Nepal (18.4 vs. 10.9%). The hypertension distribution is higher in the urban areas of all three countries. For instance, the hypertension share in urban and rural areas is 16.3 and 14.2% in India, 14.6 and 12.4% in Nepal, and 21.5 and 19.2% in Bangladesh. Strikingly, the distribution of prehypertension is higher in comparison to hypertension in urban and rural areas of all three countries.

The prevalence of prehypertension and hypertension is higher among illiterate respondents in Bangladesh (36.7 and 23.6%) and India (43.7 and 17.3%). In Nepal, hypertension is higher among respondents with a primary level of education (15.8%), whereas individuals belonging to the upper quintile of the wealth index have elevated blood pressure compared to medium and poor respondents. Respondents who are divorced, separated, or widowed have elevated blood pressure compared to those who are currently married and never married. The prevalence of prehypertension and hypertension is high among overweight and obese respondents in all three countries.

### 5.2. Prevalence of prehypertension and hypertension

**Figure 2:**
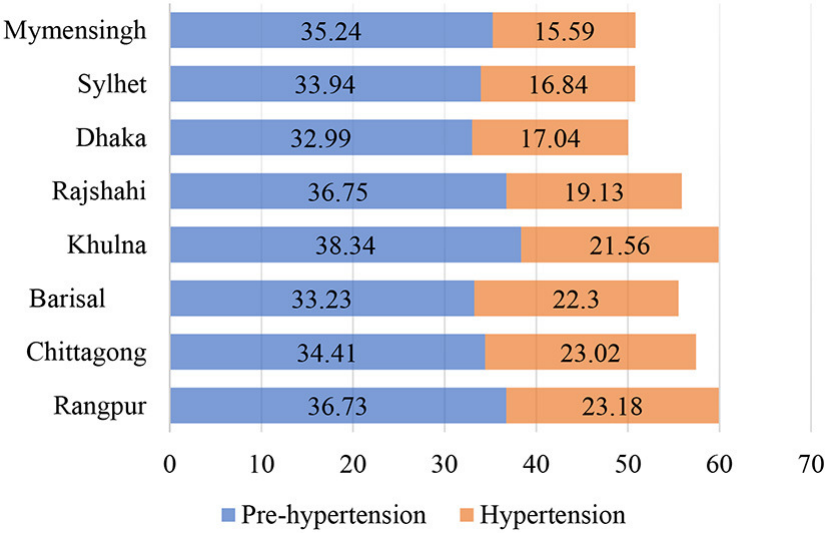
Prevalence of prehypertension and hypertension by division in Bangladesh. Source: authors' estimation from DHS data.

**Figure 3:**
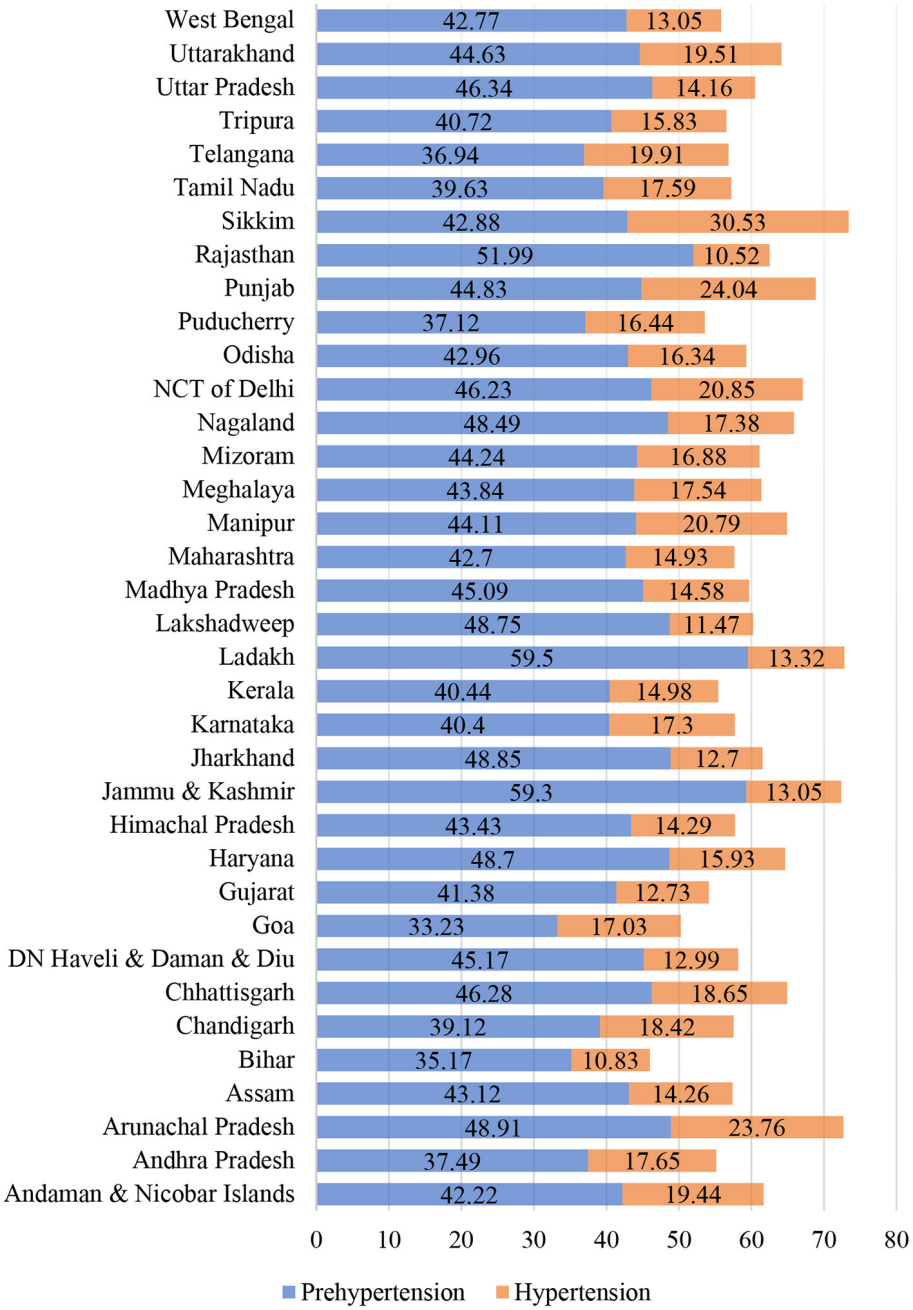
Prevalence of prehypertension and hypertension by States in India. Source: authors' estimation from DHS data.

**Figure 4:**
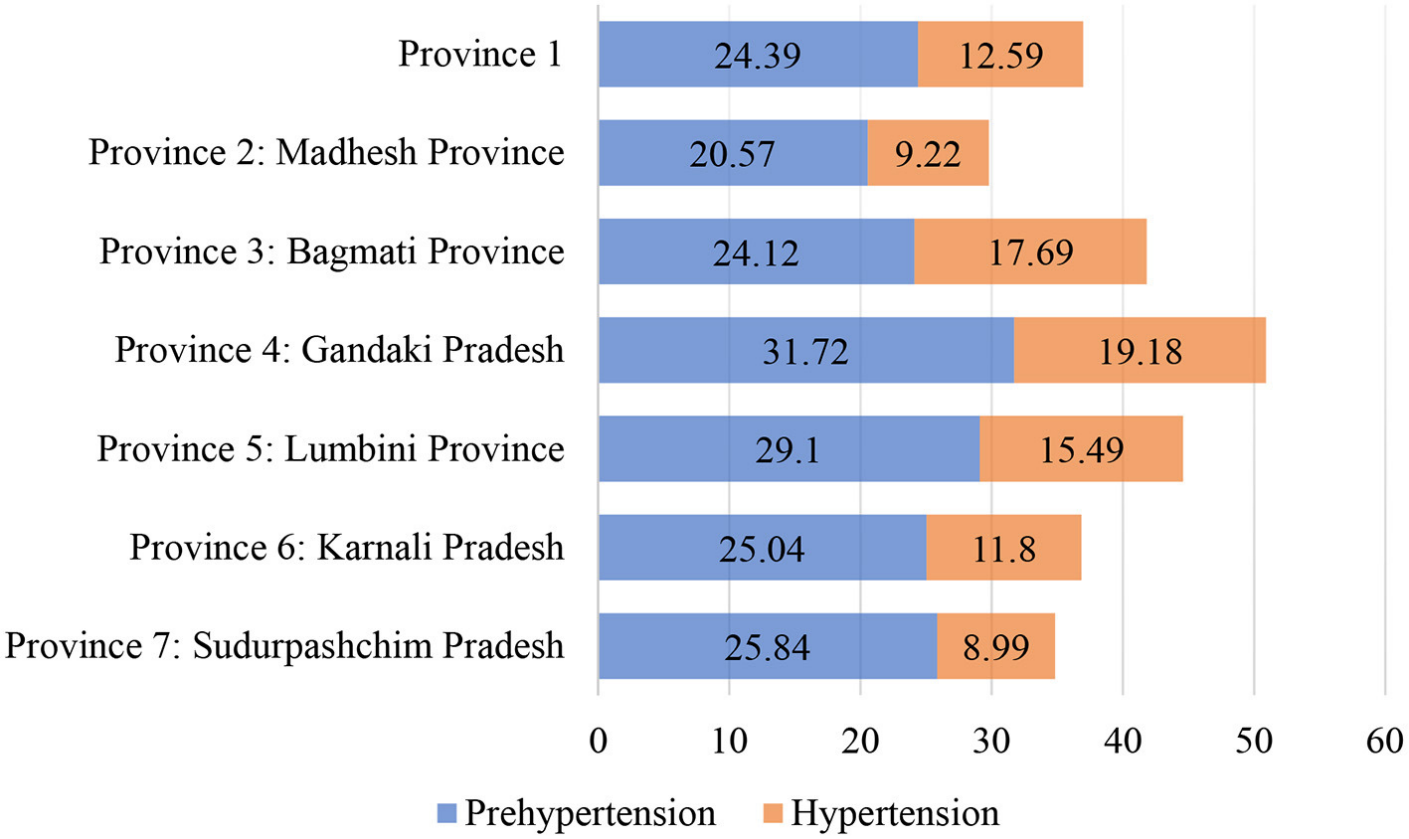
Prevalence of Prehypertension and Hypertension by Province in Nepal. Source: authors' estimation from DHS data.

Figures 2–**4** shows the prevalence of prehypertension and hypertension in the region under study. Figure 2 shows that in Bangladesh, the prevalence of prehypertension is highest in Khulna (38.34%), and Rajshahi (36.75%), and it is least in Dhaka (32.99%) and Barisal (33.23%). Almost all districts of Bangladesh have a higher prevalence of hypertension compared to all regions of Nepal and some states of India. Rangpur (23.18%) has the highest prevalence of hypertension, followed by Chittagong (23.02%) and Barisal (22.3%) among the population aged 18–49 years (see Figure 2).

Figure 3 shows that prehypertension is high in Ladakh (59.5%), Jammu and Kashmir (59.3%), and Rajasthan (52%) in the Northern region of India. From Northeastern India, Arunachal Pradesh (48.9%), Nagaland (48.5%), Mizoram (44.2%), and Manipur (44%) have a high prevalence of prehypertension. Jharkhand (49.0%) has the highest prehypertension prevalence in India's eastern region. Although the prevalence of prehypertension is relatively low in Telangana (37.0%), Bihar (35.2%), and Goa (33.2%), the rate is high enough to be of concern. In India, Sikkim (30.5%), and Arunachal (23.8%) have a higher prevalence of hypertension; Punjab (24.0%), the National Capital Territory of Delhi (20.9%), and Uttarakhand (19.5%) from the Northern region also have a higher prevalence of hypertension.

In Nepal, Province 4-Gandaki Pradesh has the highest prehypertension at 31.7%, followed by Province 5-Lumbini Province (29.1%), Province 7-Sudurpashchim Pradesh (25.84%), Province 6-Karnali Pradesh (25.04%), Province 1 (24.39%), Province 3-Bagmati Province (24.12%) and Province 2-Madhesh Province (20.57%) (Figure 4). In the case of Nepal, province 4 (Gandaki Pradesh) has the highest prevalence of hypertension with a rate of 19.18%, followed by Province 3-Bagmati (17.69%), and Province 5-Lumbini (15.49%); it is least in Province 2-Madhesh (9.22%) (see Figure 4).

### 5.3. Determinants of prehypertension and hypertension using multinomial logit regression

As the dependent variable (blood pressure: normal, prehypertension, and hypertension) is three discrete mutually exclusive, we have used multinomial logistic regression. Further, we also perform the Brant test to explore the feasibility of ordered logistic regression. The significant overall chi-square value from the Brant test suggests that ordered logit's assumptions are not met for all three models; therefore, we use multinomial logistic regression.

Table 3 presents the results from multinomial logistic regressions in terms of risk ratios, i.e., a coefficient that indicates how the risk of the outcome falling in the comparison group compared to the referent group changes. When the risk ratio is >1, it indicates that this risk increases as the variable increases. In other words, the relative risk ratio higher (or lower) than 1 suggests that the group indicated by the variable has higher (and lower, respectively) probability than the base group, with other explanatory variables remaining unchanged. Table 3 shows the associated factors related to prehypertension and hypertension in Bangladesh, India, and Nepal.

**Table 3:** Multinomial logistic regression for prehypertension and hypertension with associated factors in South Asia.

**Characteristics**	**Bangladesh (8,613)**		**India (637,396)**		**Nepal (8,924)**	
	**Prehypertension**	**Hypertension**	**Prehypertension**	**Hypertension**	**Prehypertension**	**Hypertension**
**Age (18–30 years)** ^**a**^						
31–39	1.3 (1.15, 1.47)^***^	2.81 (2.39, 3.31)^***^	1.64 (1.62, 1.67)^***^	2.49 (2.43, 2.54)^***^	1.67 (1.47, 1.91)^***^	2.56 (2.12, 3.09)^***^
40–49	1.47 (1.28, 1.7)^***^	5.11 (4.3, 6.08)^***^	2.26 (2.22, 2.3)^***^	5.61 (5.48, 5.74)^***^	2.18 (1.89,2.52)^***^	5.38 (4.44, 6.51)^***^
**Sex (male)** ^**a**^						
Female	0.66 (0.59, 0.73)^***^	0.91 (0.8, 1.04)	0.42 (0.41, 0.42)^***^	0.35 (0.34, 0.36)^***^	0.49 (0.44, 0.55)^***^	0.38 (0.33, 0.44)^***^
**Place of residence (urban)** ^**a**^						
Rural	0.96 (0.86, 1.07)	0.95 (0.83, 1.09)	0.99 (0.98, 1)	0.96 (0.94, 0.98)^***^	0.99 (0.89, 1.11)	1.0 (0.87, 1.16)
**Educational level (illiterate)** ^**a**^						
Primary	0.89 (0.76, 1.04)	1.02 (0.85, 1.23)	0.99 (0.97, 1.01)	1 (0.98, 1.03)	0.97 (0.83, 1.13)	1.21 (0.99, 1.49)^*^
Secondary	0.97 (0.82, 1.14)	1.05 (0.86, 1.29)	0.89 (0.87, 0.9)^***^	0.85 (0.83, 0.87)^***^	0.99 (0.85, 1.14)	1.18 (0.97, 1.44)^*^
Higher	1.02 (0.84, 1.23)	1.13 (0.9, 1.43)	0.83 (0.82, 0.85)^***^	0.71 (0.69, 0.74)^***^	0.99 (0.82, 1.18)	1.18 (0.92, 1.51)^***^
**Wealth index (poor)** ^**a**^						
Middle	1.12 (0.98, 1.29)^*^	1.24 (1.04, 1.48)^***^	1 (0.99, 1.02)	1.03 (1.01, 1.06)^***^	0.89 (0.77, 1.02)^*^	0.9 (0.74, 1.09)
Rich	1.05 (0.92, 1.2)	1.29 (1.09, 1.51)^***^	1.03 (1.01, 1.05)^***^	1.03 (1.0, 1.05)^**^	0.8 (0.7, 0.91)^***^	0.9 (0.76, 1.07)
**Marital status (never married)** ^**a**^						
Currently married	0.99 (0.85, 1.15)	0.85 (0.68, 1.07)	1.14 (1.12, 1.16)^***^	1.25 (1.21, 1.28)^***^	0.97 (0.83, 1.14)	1.4 (1.07, 1.84)^**^
Formerly/ever married	1.0 (0.74, 1.36)	1.03 (0.71, 1.49)	1.14 (1.1, 1.17)^***^	1.47 (1.4, 1.53)^***^	1.09 (0.76, 1.56)	1.52 (0.92, 2.5)^*^
**Body mass index (normal)** ^**(a)**^						
Overweight	2.04 (1.79, 2.33)^***^	3.9 (3.37, 4.52)^***^	1.57 (1.55, 1.6)^***^	2.5 (2.46, 2.55)^***^	2.45 (2.13, 2.82)^***^	4.23 (3.58, 5.01)^***^
Obese	3.44 (2.56, 4.62)^***^	6.14 (4.5, 8.37)^***^	1.88 (1.83, 1.93)^***^	4.31 (4.18, 4.44)^***^	3.89 (2.92, 5.17)^***^	8.63 (6.37, 11.69)^***^

*** ≤ 0.01 (99% CI);

** ≤ 0.05 (95% CI);

* ≤ 0.1 (90% CI): the model is also controlled for region variable. Confidence interval is provided in the bracket; the model was also adjusted for regions in the respective countries.

a Base category.

Source: authors' estimation from DHS Data.

The risk ratio for having prehypertension and hypertension with one unit increases in the age for a group 31–39 years relative to <30 years (base group), given that all the other variables are held constant is 1.3 (1.15, 1.47)^***^ and 2.81 (2.39, 3.31)^***^ for Bangladesh, 1.64 (1.62, 1.67)^***^ and 2.49 (2.43, 2.54)^***^ for India, and 1.67 (1.47, 1.91)^***^, and 2.56 (2.12, 3.09)^***^ for Nepal. Similarly, the risk ratio for having prehypertension and hypertension with one unit increases in the age for a group 40–49 years compared to the base group holding other factors fixed in the model is 1.47 (1.28, 1.7)^***^ and 5.11 (4.3, 6.08)^***^ for Bangladesh, 2.26 (2.22, 2.3)^***^ and 5.61 (5.48, 5.74)^***^ for India, and 2.18 (1.89, 2.52)^***^ and 5.38 (4.44, 6.51)^***^ for Nepal. These results are all significant at one percent level. It is evident from these results that elderly and economically active populations of age between 31–39 and 40–49 years have a higher likelihood of having prehypertension and hypertension in these regions.

Next, we report the results concerning gender differences in relative risk ratio comparing females to males for prehypertension and hypertension, given that the other variables are constant. Our results show that, for females relative to males, the relative risk of being prehypertensive and hypertensive in all three countries is expected to decrease by a factor of 0.66 (0.59, 0.73)^***^ and 0.91 (0.8, 1.04) in Bangladesh, 0.42 (0.41, 0.42)^***^ and 0.35 (0.34, 0.36)^***^ in India, 0.49 (0.44, 0.55)^***^ and 0.38 (0.33, 0.44)^***^ in Nepal. In other words, females are less likely to get elevated blood pressure (hypertension) than males in South Asian countries. These results are interesting because females do more household activities and physical labor than males in these countries due to the culture.

The relative risk ratio of getting hypertensive among rural residents is less likely in Bangladesh, India, and Nepal. A little can be conjectured here that prehypertension and hypertension are prevalent in urban areas where physical activities are limited and in rural areas where intensive labor is required for the people to make their daily living. Looking at these results, geographical location or regional differences explain the prevalence of hypertension, but it needs more detailed inspection.

Education levels are calculated by taking the illiterate population as a reference (base) group and comparing it with other levels of education, such as primary, secondary, and higher. Compared to the illiterate population, respondents with higher education are less likely to have prehypertension and hypertension. This result is not significant for the case of Nepal and Bangladesh. The risk ratio for prehypertension and hypertension among those with a secondary and higher level of education in India is 0.89 (0.87, 0.9)^***^, 0.85 (0.83, 0.87)^***^ and 0.83 (0.82, 0.85)^***^, 0.71 (0.69, 0.74)^***^. These results indicate that with an increase in education level, people are less likely to develop prehypertension and hypertension in India. We conjecture that education might have a country-specific effect that interacts with culture and geography. Even though these countries share several similar food habits and lifestyles, more country-specific effects might have been captured here for these differences and similarities.

Finally, we examine the risk ratios concerning wealth index, occupations, marital status, and BMI. People in the upper wealth quintile, such as middle class and wealthy (rich) compared to poor households, are more likely to develop hypertension in India, and the risk ratio is 1.03 (1.01, 1.06)^***^ and 1.03 (1.0, 1.05), holding other factors fixed. Similarly, in Bangladesh, the risk ratio of hypertension in the middle class and rich households compared to the poor is 1.24 (1.04, 1.48)^***^ and 1.29 (1.09, 1.51)^***^, holding other factors fixed. In Nepal, the wealth index is inconclusive. The risk ratio for the likelihood of prehypertension in India and Nepal for the rich household compared to the poor is 1.03 (1.01, 1.05)^***^ and 0.8 (0.7, 0.91)^***^ and significant at one percent level. The respondent's marital status affects prehypertension and hypertension in India and Nepal. The risk ratio of developing hypertension among currently married individuals and ever married (compared to never married) in India is 1.25 (1.21, 1.28) ^***^ and 1.47 (1.4, 1.53)^***^, and in Nepal, it is 1.4 (1.07, 1.84) ^**^ and 1.52 (0.92, 2.5)^*^, holding other factors fixed.

The currently married and ever-married respondents are more at risk of developing prehypertension and hypertension than the never-married ones, which may be related to culture, age, and lifestyle among married individuals. In South Asia, overweight and obese people are at high risk of developing prehypertension and hypertension. It is widely known that obese individuals have greater chances of being hypertensive and developing CVDs.

The relative risk ratio for developing prehypertension among respondents who are overweight is 2.04 (1.79, 2.33)^***^ in Bangladesh, 1.57 (1.55, 1.6)^***^ in India, and 2.45 (2.13, 2.82)^***^ in Nepal. Similarly, for those who are obese, the relative risk ratio of developing prehypertension is 3.44 (2.56, 4.62)^***^ in Bangladesh, 1.88 (1.83, 1.93)^***^ in India, and 3.89 (2.92, 5.17)^***^ in Nepal. At the same time, the risk of developing hypertension among the population suffering from obesity when holding other factors fixed is 6.14 (4.5, 8.37)^***^ in Bangladesh, 4.31 (4.18, 4.44)^***^ in India, and 8.63 (6.37, 11.69)^***^ in Nepal. Overall, we have identified several similar trends and some differences between countries and within countries. Indicators responsible for prehypertension and hypertension in South Asia are educated individuals, a population with a rich and middle wealth index, higher education, ever-married, and high BMI. A striking finding is that even younger adults are at higher risk of prehypertension and hypertension.

## 6. Discussion

The burdens of non-communicable diseases in South Asia vary according to the country's development, geographical locations, and the development of epidemiological transition (25). This study shows the high burdens of hypertension and prehypertension in the productive mid-life population, which might adversely affect workforce productivity and economic development in South Asian countries like Bangladesh, India, and Nepal (23, 31). The geographical disparity shows that mainly the eastern region of India, including Bangladesh and a few regions of Nepal, have a cluster of prehypertension and hypertension (32). Adhikari et al. (33) report that the prevalence of hypertension is as high as 70% in Nepal and up to 55% in Karnali (rural area) and Bagmati province (urban area). Interestingly, we can see that hypertension and prehypertension are prevalent in poor (rural areas) and rich (urban areas), such as the capital city of Nepal, Kathmandu. Prehypertension and hypertension are likely to increase with age, poverty, and inequality (34). The poorest and underprivileged groups, compared to rich ones, have a 1.6 times higher prevalence of prehypertension and hypertension; thus, the Health Technology Assessment (HTA) program and interventions should be strengthened and evaluated with economic evaluation (35). Especially in urban areas, prehypertension needs a particular focus as it can be prevented through simple changes in lifestyle and diet. It is evident from the country-specific studies from the South Asian regions that the prevalence of hypertension is high among residents of urban areas and those from more affluent backgrounds (5, 36–38).

This study shows a positive association between hypertension and prehypertension with gender (male) in India, Nepal, and Bangladesh. Females are less likely to develop prehypertension and hypertension in comparison to their male counterparts in all three countries. Other studies from Bangladesh, India, and Nepal, have similar findings (39, 40). Similarly, Agho et al. (19) suggest that prehypertension and hypertension are higher in males than females. In one study in Sri Lanka, authors find that low birth weight and age are significant factors affecting hypertension (32). Few studies contradict the facts about the relationship between gender and hypertension (5). In Bangladesh and Nepal, residents from urban areas are at elevated risk of developing hypertension (41, 42). This may be due to a sedentary lifestyle, unhealthy diet, and pollution (41). With an increase in education level, respondents are at higher risk of prehypertension and hypertension in Nepal and Bangladesh (5, 41). The reason behind this finding might be that with education, there are higher income and lifestyle changes, which might become one source of the prevalence of prehypertension and hypertension. However, the pattern is the opposite in India: with an increase in education level, prehypertension and hypertension were less likely (38). Overall, gender, age, and education appeared as independent risk factors for prehypertension and hypertension. Clearly, females in South Asian regions do several household chores more frequently than males and are also less likely to be involved in activities such as alcohol consumption and smoking.

Developing countries have been urbanizing rapidly and show a positive relationship between urbanization and the CVD burden (43). Previous studies have shown a geographical disparity playing a significant role in the prevalence of hypertension in South Asian countries (38, 41). Mishra et al. (42) and Prenissl et al. (44) also report that adults with higher income levels have hypertension and these trends are similar in India, Nepal, and Bangladesh. Married respondents are less likely to develop prehypertension and hypertension (13), whereas widowed, divorced, and separated respondents are more at risk of prehypertension and hypertension. A study shows that widowed/divorced females are at higher risk for cardiovascular diseases and are less inclined to seek treatment (39, 42). Hasan et al. (13) and Iqbal et al. (39) find that overweight and obese adults are at higher risk of developing prehypertension and hypertension. Similarly, we also find that with an increase in weight, the risk of hypertension and prehypertension increases in all these three countries' study areas. The risk of having prehypertension among the overweight and obese is higher; thus, increasing weight shall be one of the underlying causes of hypertension among adults. Adults with prehypertension are at a higher risk of developing hypertension. Moreover, with the ongoing changing lifestyle, lack of physical activity, and unhealthy diet, the adult population is equally at risk as the elderly. Another important observation is that the burden of non-communicable diseases (NCDs) and hypertension in various socioeconomic groups in South Asian countries have increased over the decades (45).

By examining the prevalence and determinants of hypertension and prehypertension together in a single framework, this study provides unique insight for governments, medical societies, and non-governmental organizations to create a support system to promote preventive programs aiming to stop prehypertension from becoming hypertension through increasing public awareness and policy interventions. However, future studies should also look into whether and how the consumption of unhealthy products, i.e., sugar-sweetened food, physical activity, nutritional food intake, and bad habits like smoking and alcohol drinking, would affect CVDs. Also, more studies are required on policy and behavioral interventions, such as nudging public behaviors. Several past studies have tried to control hypertension with one-way regulation models, like increasing taxes on alcohol, tobacco consumption, and SSBs.

This study has identified factors and prevalence of prehypertension and hypertension; thus, hypertension can be controlled at an earlier stage. Notably, some factors relating to prehypertension are modifiable and can be addressed with appropriate policy transformations. India has taken interventions such as the India Hypertension Control Initiative (IHCI) and set a target to relatively reduce 25% in the prevalence of hypertension by 2025. Similarly, Bangladesh has also given particular attention to minimizing the burden of non-communicable diseases found among the poor populations in rural areas and the wealthy in urban areas. Furthermore, this study suggests urgently setting intervention programs at multiple levels to increase hypertension awareness; such interventions are access to high blood pressure treatment *via* community-wide health programs and community-level non-pharmacologic interventions, like weight loss and sodium reduction campaigns or home blood pressure measuring programs. These cheap community driven interventions can effectively help reduce high blood pressure levels and identify the need for drug treatment in patients with hypertension. Other effective programs include self-monitoring of blood pressure levels; such interventions have become possible due to access to affordable advanced digital technology, and it is easy to manage by individuals.

## 7. Conclusion

This study has empirically analyzed the determinants of prehypertension and hypertension together by looking at socio-demographic characteristics in a single framework utilizing the recent extensive sample data from Bangladesh, India, and Nepal. The study shows that geographical locations (division in Bangladesh, states in India, and province in Nepal), gender differences, level of education only in the case of India, wealth, and overweight and obesity are important determinants of the prevalence of hypertension and prehypertension. Most importantly, the patterns for prehypertension follow a similar trend in these countries, such that prehypertension is highly prevalent in the young and economically active age group. In this way, understanding the prevalence of prehypertension in South Asia and its alarming rate mandates preventive measures. The prevalence of hypertension and prehypertension and the disease burdens in the productive mid-life period shall adversely affect workforce productivity and economic development in these regions.

Concurrently, an earlier understanding of mechanisms can prevent precise estimation of the size of NCDs burdens, such as hypertension and prehypertension. The necessary change in direction is evident as the burden is rising. Thus awareness, treatment, and control of prehypertension have colossal potential to reduce the number of hypertension cases. In many middle- and low-income countries, prehypertension, and hypertension have increased rapidly. The magnitude of the problem is large enough to demand urgent attention and action to inform policy better and to monitor change. Proven solutions to the challenges of hypertension can be adjusted to meet the needs of individuals and communities with different healthcare infrastructures and resources. Enhanced efforts to prevent and treat hypertension are attainable. Pursuing cost-effective approaches that yield substantial health benefits should be a very high priority for societies and the public health community, together with some behavioral interventions to further introduce evidence-based policy.

## Data availability statement

Publicly available datasets were analyzed in this study. This data can be found here: https://dhsprogram.com/.

## Author contributions

DR and RM contributed the conceptualization of the research idea, data cleaning, analysis, write-up, editing, and structuring. RT and TS contributed to the conceptualization of the research idea, write-up, editing, and structuring. All authors contributed to the article and approved the submitted version.

## Footnotes

^1^Epidemiologic transition describes the changing patterns of mortality, population age distributions, fertility, life expectancy, and causes of death (8). It is generally believed that epidemiologic transitions prior to the 20th century were closely associated with rising living standards, nutrition, and sanitation.
